# Ambient Temperature and Cardiac Biomarkers: A Meta-Analysis

**DOI:** 10.2174/1573403X19666230804095744

**Published:** 2023-10-02

**Authors:** Muhammad Ismail Khan, Zafar Rasheed

**Affiliations:** 1 Faculty of Medicine, School of Public Health, University of Queensland, Brisbane, Australia;; 2 Department of Pathology, College of Medicine, Buraidah, Qassim University, Buraidah, Saudi Arabia

**Keywords:** Meta-analysis, ambient temperature, cardiac markers, c-reactive protein, blood pressure, low-densitylipoprotein

## Abstract

This study quantified the effect of cold or heat exposure of ambient temperature on the alteration of well-known cardiac markers. A meta-analysis was performed using the PRISMA guidelines. Peer-reviewed studies on ambient temperature and cardiac biomarkers were retrieved from MEDLINE, ScienceDirect and Google Scholar from January 2000 to February 2022. The pooled effect sizes of ambient temperature on cardiac biomarkers c-reactive protein, soluble-cell adhesion-molecule-1, soluble-intercellular-adhesion-molecule-1, total cholesterol, low-density-lipoprotein, interleukin-6, B-type-Natriuretic-Peptide; systolic/diastolic blood pressure were quantified using a random-effects meta-analysis. A total of 26 articles were included in the meta-analysis after screening the titles, abstracts and full texts. The pooled results for a 1°C decrease of ambient temperature showed an increase of 0.31% (95% CI= 0.26 to 0.38) in cardiac biomarkers (p=0.00; I-squared=99.2%; Cochran’s Q=5636.8). In contrast, the pooled results for a 1°C increase in ambient temperature showed an increase of 2.03% (95% CI= 1.08 to 3.82) in cardiac biomarkers (p=0.00; I-squared=95.7%; Cochran’s Q=235.2). In the cardiovascular (CV) population, the percent increase in cardiac biomarkers levels due to a decrease/increase in ambient temperature was greater. This study showed the decrease/increase in ambient temperature has a direct correlation with the alterations in cardiac biomarkers. These findings are useful for managing temperature-associated cardiovascular mortality.

**Registration:**

This meta-analysis is registered on the National Institute for Health and Care Research (NIHCR) for the PROSPERO with registration ID CRD42022320505.

## 1 INTRODUCTION

Extreme temperatures have always been a major public health concern, and the evaluation of the impact of ambient temperatures on the risk of cardiovascular (CV) mortality has received more attention in recent years [1, 2]. Yet, none of the meta-analyses evaluate the effect of ambient temperature on alterations in cardiac markers. Low temperatures have been connected to heart failure hospitalizations and the occurrence of cardiovascular events [3, 4]. However, during hot weather, there has been an increase in morbidity and mortality [5]. It's likely that the molecular pathways that support cardiovascular health outcomes change throughout the year, depending on the ambient outside temperature. However, a few studies have examined these connections in heart failure patients [6, 7]. Furthermore, it is unknown if short-term changes in weather patterns are linked to preclinical changes in heart failure conditions [8]. The known cardiovascular markers such as c-reactive protein (CRP), systolic/diastolic blood pressure, total cholesterol, low-density lipoprotein (LDL), interleukin-6 (IL-6), soluble cell adhesion molecule-1 (sCAM-1), soluble intercellular adhesion molecule-1 (sIAM-1) are indicators of systemic inflammation, immunological function, and ventricular remodelling, have been linked to increased morbidity and death in heart failure patients [9-13]. Moreover, the B-type natriuretic peptide (BNP), a neurohormone produced mostly by the heart's ventricles in response to increased wall stress, has also been proven to be a biomarker of sudden death in people with chronic heart failure [14]. In experimental research involving healthy young adult individuals, changes in heart rate variability have been linked to both acute air temperature drops and increases [15, 16]. Individuals with underlying cardiovascular disease, such as cardiac rehabilitation patients and the elderly, may be more susceptible to temperature changes because of their underlying disease [17, 18]. The analysis of a panel of biomarkers may reveal information about the underlying pathophysiology of a disease as well as specific therapy responses [19]. Some studies have found that cardiac biomarkers are significantly greater in the winter than in the summer, implying a link between outdoor temperature and cardiac biomarkers. Other research, on the other hand, has shown no link between temperature and cardiac biomarkers. Small research populations hampered the ability of several of these investigations to detect a link between temperature and cardiac biomarkers. As a result, we aggregated evidence from relevant epidemiological studies to investigate the link between ambient temperature and cardiac biomarkers in this systematic review and meta-analysis.

## 2 MATERIALS AND METHODS

### 2.1 PRISMA Guidelines

The updated guidelines for the preferred reporting items for systematic reviews and meta-analyses (PRISMA) [20] were used for the preparation of this article. This meta-analysis is registered on the National Institute for Health and Care Research (NIHCR) for the PROSPERO with registration ID CRD42022320505.

### 2.2 Keywords and Terms used in the Database for Literature Search

A systematic literature search was performed in two stages. In stage one literature search, the keywords used were ambient temperature; cardiac markers; cold temperature; hot temperature. All sub-terms related to these keywords were used, but the search was limited to human studies in English only. In the second stage of the literature search, names of well-known cardiac markers were used, such as C-Reactive Protein (CRP); Soluble Cell Adhesion Molecule-1 (sVCAM); Soluble Intercellular Adhesion Molecule-1 (sIAM); Total cholesterol (TC); low-density lipoprotein cholesterol (LDL-C); Interleukin-6 (IL-6); B-type Natriuretic Peptide (BNP); Systolic blood pressure (SBP); diastolic blood pressure (DBP).

### 2.3 Strategy of Literature Search and Studies Eligibility Criteria

Only peer-reviewed published studies on ambient temperature and cardiac markers were retrieved from MEDLINE, Science Direct, and Google Scholar from January 2000 to February 2022. Reference lists in the selected studies were thoroughly examined to check for studies missed in our database searches. The primary inclusion criteria of studies selection were studies on humans and were published in English. Studies meeting these primary inclusion criteria with search keywords were initially reviewed for the titles and abstracts. The studies that met initial screening criteria were reviewed. Based on the temperature exposure used in the studies, the identified studies were categorized into two groups: the cold temperature exposure and the hot temperature exposure. The eligibility criteria of the studies was that the studies must be population-based; studies must provide a quantitative correlation between ambient temperature and cardiac markers, outcome of the studies must present the alterations in cardiac markers quantitatively. All non-population-based studies, such as studies performed on animals, and cells were excluded. Moreover, studies reporting only qualitative measurements of temperature and alterations in cardiac markers were also excluded. The selection criteria of the studies are summarized in Fig. (**1**).

### 2.4 Selected Studies Quality Check and Data Extraction

The Critical Appraisal Skills Program for systematic review [21] was used to assess the quality of all selected studies. In the CASP checklist for systematic review, the quality score ranged from 1 to 10. A cut-off point of 6 was used for any study to be included in the meta-analysis. The quality of studies was assessed on the study design and timespan; the population of study; sample size; statistical methods; main temperature exposure variable; confounder variables, and lag time. The data were extracted from selected studies, including the name of the research group, publication year, study period, study location, study population, any lag time used, study type, population size, temperature exposure cold or hot, name of cardiac marker and final outcome on the percent increase in cardiac markers with 95% confidence interval (CI).

### 2.5 Data Analysis

The data were analyzed in the form of pooled effect size which was computed from the dataset of cold and hot temperature exposure on the alterations in cardiac markers using the random-effect model and presented as forest plots. All estimates were standardized to the percent increase in cardiac markers per 1°C decrease or increase in ambient temperature. If the information in an article was incomplete to convert estimates, the article was excluded. The random-effect model allows us to check the heterogeneity among the selected studies. Statistics of inconsistency, I-squared (I2) values were reported that showed the estimated percent of the total variance, which explained the heterogeneity among the studies, and I2> 50% demonstrates a statistically significant heterogeneity. We also conducted a series of sensitivity analyses to test whether the meta-estimates were reliant on the inclusion of one influential study. For each analysis, we compared the meta-estimates before and after removing a single study with the largest and smallest weight. All meta-analyses were conducted using the MetaXL feature of Microsoft Excel 2010.

## 3 RESULTS

**Fig. (1):**
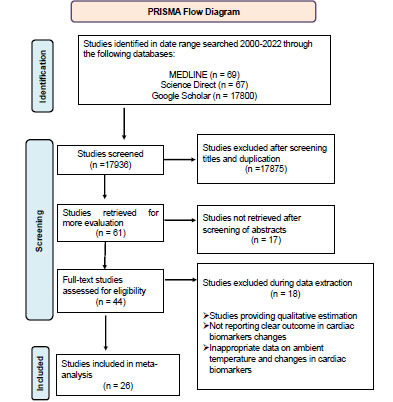
Preferred reporting items for systematic reviews and meta-analysis (PRISMA) flow diagram for the selection of studies.

Our comprehensive search resulted in the acquisition of 17,936 articles. After reviewing the title and removing duplicates, 17875 were eliminated, 17 not needing further screening after reviewing the abstract and eighteen were excluded during data extraction due to not providing qualitative estimation and not reporting clear outcomes in terms of cardiac biomarkers. In the end, a total of 26 articles were included in the meta-analysis after screening (Fig. **1**). Studies showing the alterations in the known cardiac biomarkers due to the variations in ambient temperature were included. The meta-analysis was performed with the following cardiac biomarkers c-reactive protein [3, 22-28], soluble cell adhesion molecule-1 [27], soluble intercellular adhesion molecule-1 [27], total cholesterol [24], low-density lipoprotein cholesterol [24, 27, 29], interleukin-6 [24, 28], fibrinogen [28], B-type natriuretic peptide [3], Vitamin D [24], Systolic blood pressure [23, 24, 30-45] and diastolic blood pressure [23, 30-37, 41, 44-47]. The complete characteristic details of the included studies in the meta-analysis are summarized in Table **1**. Of the twenty-six studies included, twenty-one were cohort, two were cross-sectional, two retrospective, and one was a baseline survey. The temperature measurement method employed in each of the investigations was different. Twenty-five studies used outside temperature data from meteorological monitoring stations near the study locations to investigate the association between outdoor temperature and cardiac biomarkers. The most common study population was adults, three studies looked at elderly patients, five looked at hypertensive patients, and one looked at type 2 diabetics who were older. Nine studies were conducted in China, six in the US, and the remainder in Japan, the United Kingdom, Germany, France, Korea, and the Netherlands.

**Fig. (4):**
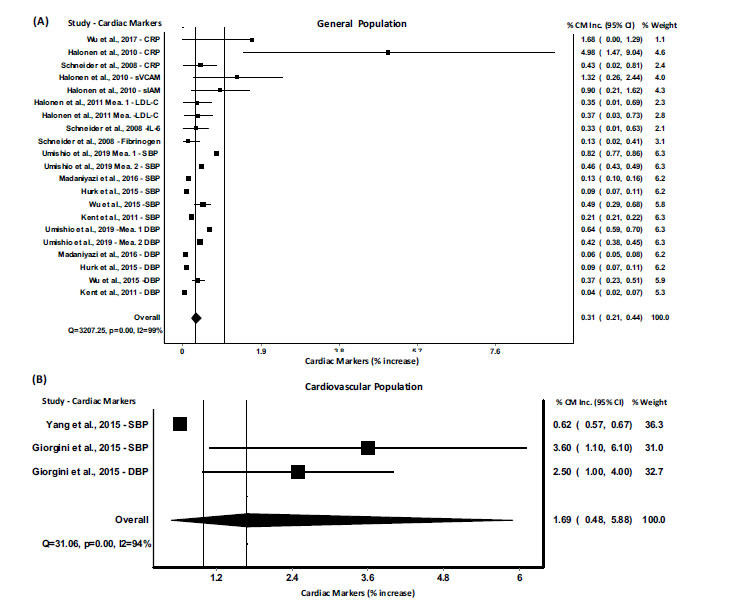
Meta-analysis of alterations in the cardiac markers with a decrease of 1°C in ambient temperature in general (**A**) and cardiovascular (CV) (**B**) population. All estimates were standardized to the percent increase in cardiac markers per 1°C decrease in ambient temperature. **Abbreviations:** CM, cardiac markers; CRP, C-Reactive Protein; sVCAM, Soluble Cell Adhesion Molecule-1; sIAM, Soluble Intercellular Adhesion Molecule-1; TC, Total cholesterol; LDL, low-density lipoprotein cholesterol; IL-6, Interkleukin-6; BNP, B-type Natriuretic Peptide; SBP, Systolic blood pressure; DBP, diastolic blood pressure; Mea, measurement.

**Fig. (5):**
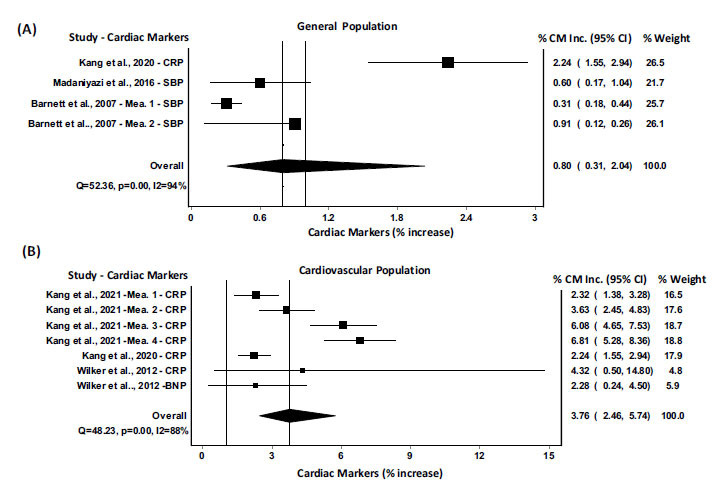
Meta-analysis of alterations in the cardiac markers with an increase of 1°C in ambient temperature in general (**A**) and cardiovascular (**B**) populations. All estimates were standardized to the percent increase in cardiac markers per 1°C decrease or increase in ambient temperature. **Abbreviations:** CM, cardiac markers; CRP, C-Reactive Protein; BNP, B-type Natriuretic Peptide; SBP, Systolic blood pressure; Mea, measurement.

Low- and high-temperature exposure impact sizes were reported separately in Figs. (**2** and **3**), respectively. A total of 26 articles were included in the meta-analysis after screening the titles, abstracts, and full texts. The complete meta-analysis of all included studies with cardiac markers CRP, sVCAM, LDL, IL-6, fibrinogen, BNP, SBP, DBP are summarized in Fig. (**2**), and the pooled results for a 1°C decrease of ambient temperature showed an increase of 0.31% (95% CI= 0.26 to 0.38) in cardiac biomarkers (p=0.00; I-squared=99.2%; Cochran’s Q=5636.8). Whereas, Fig. (**3**) summaries the pooled results indicating that a 1°C increase in ambient temperature resulted in an increase of 2.03% (95% CI= 1.08 to 3.82) in cardiac biomarkers CRP, BNP, SBP, and DBP (p=0.00; I-squared=95.7%; Cochran’s Q=235.2). In order to determine the role of the decrease or increase of ambient temperature among individuals having cardiovascular problems, a meta-analysis was performed among the cardiovascular population, and the results were compared with the meta-analysis of the general population. The pooled results for a 1°C decrease of ambient temperature in general population showed an increase of 0.31% (95% CI= 0.21 to 0.44) in cardiac biomarkers (p=0.00; I-squared=99%; Cochran’s Q=3207.25) (Fig. **4A**). Whereas, the pooled results for a 1°C decrease of ambient temperature in cardiovascular (CV) population showed an increase of 1.69% (95% CI= 0.48 to 5.88) in cardiac biomarkers (p=0.00; I-squared=94%; Cochran’s Q=31.06) (Fig. **4B**). On the other hand, the pooled results for a 1°C increase of ambient temperature in general population showed an increase of 0.80% (95% CI= 0.31 to 2.04) in cardiac biomarkers (p=0.00; I-squared=94%; Cochran’s Q=52.36) (Fig. **5A**). Whereas, the pooled results for a 1°C increase of ambient temperature in cardiovascular population showed an increase of 3.76% (95% CI= 2.46 to 5.74) in cardiac biomarkers (p=0.00; I-squared=88%; Cochran’s Q=48.23) (Fig. **5B**). These results indicated that the alteration in cardiac markers was greater in cardiovascular patients as compared with the general population (Figs. **4** and **5**).

## 4 DISCUSSION

To the best of our knowledge, this is the first meta-analysis that investigated the relationship between ambient temperature and cardiac biomarkers. Overall, we observed a consistent, statically significant association of both low and high temperatures with a percent increase in cardiac biomarkers levels. In the developed world, myocardial infarction (MI) is the leading cause of death, and cardiac biomarkers play a life-saving role in diagnosis, reduction of risk, controlling and making clinical decisions for the management of individuals with cardiovascular complications [48]. It is now well established that the quantification of c-reactive protein (CRP) has emerged as one of the most powerful indicators of cardiovascular risk management in a broad spectrum of patient populations [49, 50]. The standardized high-sensitivity CRP (hs-CRP) assays are now in use and have become powerful detectors to detect even minor variations in CRP below the limit of standard assays [49, 51]. Importantly, studies showed up to 2-4-fold increased cardiovascular risk in healthy individuals with elevated CRP levels [50]. In the cardiovascular population and individuals undergoing percutaneous coronary interventions, CRP levels predict both the short and long-term prognosis, identifying subgroups of individuals at high risk of recurrent events [49, 52].

Moreover, elevated levels of hs-CRP are also useful in targeting therapy at both primary and secondary preventive levels by categorizing patients' populations who would most benefit from medications [49, 53]. Similarly, B-type natriuretic peptide (BNP), soluble cell adhesion molecule-1 (sVCAM), soluble intercellular adhesion molecule-1 (sIAM), total cholesterol, LDL, IL-6, fibrinogen, systolic and diastolic blood pressure all have now been well considered as biomarkers for the management of cardiovascular complications [12, 54-56]. In view of the clinical implications of these cardiac markers, the studies on CPR levels, BNP, sVCAM, sIAM, total cholesterol, LDL, IL-6, fibrinogen, systolic and diastolic blood pressure in association with ambient temperature were included in this meta-analysis. Due to the availability of limited published studies on these cardiac markers in relation to ambient temperature, the data presented in this meta-analysis is a pool of data of all cardiac markers.

At present, the mechanism(s) underlying the relationship between these cardiac biomarkers and ambient temperature are not fully understood and many features associated with this relationship remain to be explored. Findings from the current meta-analysis provide clues that ambient temperature may affect cardiovascular morbidity and mortality. This can also be applied for the prevention and treatment of individuals susceptible to myocardial infarction. Seasonal variation in outdoor temperature can affect cardiac biomarkers and should be considered for the treatment of patients with cardiovascular diseases [57]. For instance, cardiovascular patients may require higher doses of medication in winter than at any other time of the year [58]. Besides, our findings are the scientific basis for the health effects of weather forecasts. When the temperature drops or rises sharply, the public, especially the elderly and other temperature-vulnerable groups, should be informed of appropriate precautions against the impending risk posed by sharp changes in ambient temperature. The included studies examined that the low ambient temperature is associated with increase in cardiac biomarkers. Inconsistency in the magnitude of effect sizes is due to differences between individual studies, study design, temperature measurement method, geographical location, demographic characteristics, and different temperature lags. Underlying biological mechanisms behind cold and warm temperatures – cardiac biomarkers association is still not clear. Cold stress leads to activation of the sympathetic nervous system, which in turn induces circulatory vasoconstriction [59, 60]. In response to direct exposure to cold, the renin-angiotensin-aldosterone and sympathetic system get activated [61]. These cold temperature events consequently lead to vasoconstriction and activation of neuroendocrine systems bringing about an increase in cardiac biomarkers [38, 61, 62]. On the other hand, high temperatures are also reported to alter the levels of cardiac markers, such as blood pressure in individuals with arteriolar vasodilation [63]. These mechanisms may define why individuals with cardiovascular complications are more susceptible to changes in ambient temperature. In order to investigate more on this issue, we determined the role of ambient temperature low or high among individuals having cardiovascular problems, the obtained meta-analysis data were compared with the general population. The pooled data of a percent increase in the levels of cardiac markers per 1°C reduction of ambient temperature was markedly high in the cardiovascular population compared to the pool percent increase of cardiac markers in the general population. Interestingly, a similar pattern on cardiac markers was also observed for a 1°C increase in ambient temperature in the cardiovascular population compared with the general population. These findings indicated that individuals with cardiovascular complications are more susceptible to ambient temperature changes than the general population. These meta-analysis findings have also been well supported by the fact that individuals with cardiovascular complications have been associated with endothelial dysfunctioning, which results in the weakening of their vasodilatation against the alterations in environmental temperature [40]. All these findings strongly suggested that people with a high risk of cardiovascular disorders required a regular checkup of their cardiovascular biomarkers in extremely hot or cold weather conditions.

### 4.1 Strengths and Limitations

This is the first meta-analysis to the best of our knowledge that quantitatively summarizes the effects of ambient temperature on the alteration in cardiac biomarkers. This meta-analysis has clear inclusion and exclusion criteria for the selection of studies, and we used the Newcastle–Ottawa scale to assess the quality of the study. Robustness of our finding was examined by sensitivity analysis, ensuring validity of the results. The impact of this meta-analysis is useful in both public health and clinical sections for the management of cardiovascular complications. Moreover, this meta-analysis also provides future research directions.

This meta-analysis has a few limitations. The first and most obvious limitation is the high heterogeneity that we found between the studies included. This only allows us to use a random effect model for quantification of the pool data with high heterogeneities. The second obvious limitation is the availability of the limited related studies. Due to this, we failed to perform a subgroup analysis. Specifically, we failed to do subgroup analysis due to limited studies on cardiac biomarkers such as CRP, sVCAM, sIAM, LDL, IL-6, fibrinogen, and others.

## CONCLUSION

This meta-analysis reported consistent, significant associations between ambient temperature and cardiac biomarkers, particularly c-reactive protein, LDL-cholesterol, and systolic and diastolic blood pressure. This meta-analysis found that both decreased or increased ambient temperature cause alterations in cardiac biomarkers levels. Individuals with cardiovascular complications are more susceptible to changes in environmental ambient temperature. The findings indicate that improving house insulation is required to prevent cardiac biomarkers alterations in extreme weather conditions. Especially individuals with cardiovascular complications need more intensive medical care to monitor the normal levels of cardiac biomarkers, mainly in extreme weather conditions. Future research concerning the association of environmental ambient temperature with cardiac biomarkers among a population with affordable size may lead to a better understanding of the relationship between ambient temperature and cardiac biomarkers.

## Figures and Tables

**Fig. (2):**
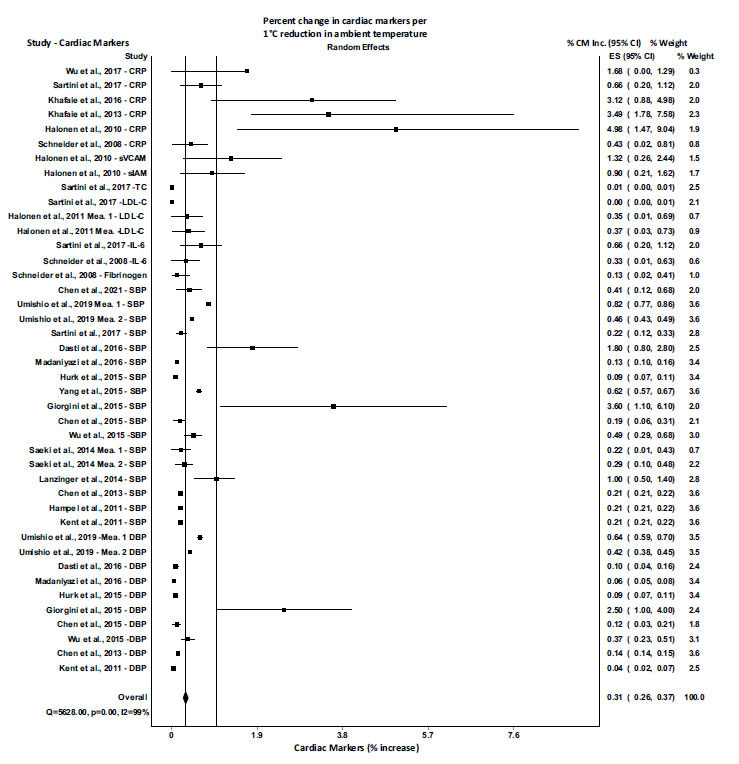
Meta-analysis of alterations in the cardiac markers with a decrease of 1°C in ambient temperature. All estimates were standardized to the percent increase in cardiac markers per 1°C decrease in ambient temperature. **Abbreviations:** CM, cardiac markers; CRP, C-Reactive Protein; sVCAM, Soluble Cell Adhesion Molecule-1; sIAM, Soluble Intercellular Adhesion Molecule-1; TC, Total cholesterol; LDL, low-density lipoprotein cholesterol; IL-6, Interkleukin-6; BNP, B-type Natriuretic Peptide; SBP, Systolic blood pressure; DBP, diastolic blood pressure; Mea, measurement.

**Fig. (3):**
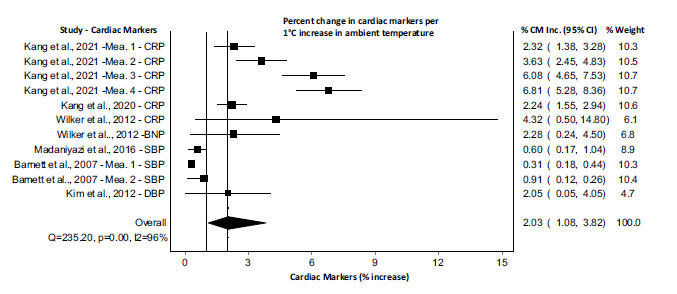
Meta-analysis of alterations in the cardiac markers with an increase of 1°C in ambient temperature. All estimates were standardized to the percent increase in cardiac markers per 1°C increase in ambient temperature. **Abbreviations:** CM, cardiac markers; CRP, C-Reactive Protein; BNP, B-type Natriuretic Peptide; SBP, Systolic blood pressure; DBP, diastolic blood pressure; Mea, measurement.

**Table 1:** Summary of studies included in the meta-analysis.

**Research** **Group**	**Year of** **Pub.**	**Study** **Period**	**Location**	**Study** **Population**	**Lag Time,** **Days**	**Study Type**	**Population** **Size**	**Temp.** **Exposure**	**Cardiac** **Marker**	**Temp** **Change, °C**	**Final Outcome on** **Cardiac Markers**
-	-	-	-	-	-	-	-	-	-	-	% Increase (95% CI)^#^
Kang *et al.*	2021	2009-2010	China	Cardiovascular	0	Cross-sectional	11,623	Outdoor	CRP	10°C Inc.	23.20 (10.38-32.80)
Kang *et al.*	2021	2009-2010	China	Cardiovascular	0	Cross-sectional	11,623	Outdoor	CRP	10°C Inc.	36.30 (24.5-48.30)
Kang *et al.*	2021	2009-2010	China	Cardiovascular	0	Cross-sectional	11,623	Outdoor	CRP	10°C Inc.	60.80 (46.5-77.30)
Kang *et al.*	2021	2009-2010	China	Cardiovascular	0	Cross-sectional	11,623	Outdoor	CRP	10°C Inc.	68.10 (52.8-83.60)
Kang *et al.*	2020	-	China	General	1-7	Cohort	11,623	Outdoor	CRP	10°C Inc.	22.40 (15.52-29.35)
Wu *et al.*	2017	2010-2011	China	General	0-2	Cohort	-	Outdoor	CRP	10 °C Dec.	1.68 (0.002-1.29)
Sartini *et al.*	2017	1997–2000	UK	Aged	0	Cohort	10056	Outdoor	CRP	5 °C Dec.	0.66 (0.20-1.12
Khafaie *et al.*	2016	2005-2007	India	Type 2 Diabetic	0-1	Cohort	1,700	Outdoor	CRP	5 °C Dec.	3.12 (0.884-4.976)
Khafaie *et al.*	2013	2005-2007	India	Type 2 Diabetic	0-7	Cross-sectional	1,392	Outdoor	CRP	1 °C Dec.	3.49 (1.78-7.58)
Wilker *et al.*	2012	-	USA	Cardiovascular	1-4	Cohort	100	Apparent	CRP	5°C Inc.	4.32 (0.50-14.80)
Halonen *et al.*	2010	2000-2008	USA	General	0-1	Cohort	673	Outdoor	CRP	5°C Dec.	4.98 (1.472-9.04)
Schneider *et al.*	2008	-	Germany	General	0-3	Cohort	5813	Outdoor	CRP	10°C Dec.	0.43 (0.02-0.81)
Halonen *et al.*	2010	2000-2008	USA	General	0-1	Cohort	673	Outdoor	sVCAM	5°C Dec.	6.6 (1.31-12.2)
Halonen *et al.*	2010	2000-2008	USA	General	0-1	Cohort	673	Outdoor	sIAM	5°C Dec.	4.52 (1.05-8.10)
Sartini *et al.*	2017	1997–2000	UK	Aged	0	Cohort	10056	Outdoor	TC	5°C Dec.	0.04 (0.02-0.07)
Sartini *et al.*	2017	1997–2000	UK	Aged	0	Cohort	10056	Outdoor	LDL	5°C Dec.	0.02 (0.01-0.05)
Halonen *et al.*	2011	1995-2008	USA	General	0-1	Cohort	478	Outdoor	LDL	5°C Dec.	1.74 (0.07-3.44)
Halonen *et al.*	2011	1995-2008	USA	General	0-2	Cohort	478	Outdoor	LDL	5°C Dec.	1.87 (0.14-3.63)
Sartini *et al.*	2017	1997–2000	UK	Aged	0	Cohort	10056	Outdoor	IL-6	5°C Dec.	3.30 (1.0-5.6)
Schneider *et al.*	2008	-	Germany	General	0-3	Cohort	5813	Outdoor	IL-6	10°C Dec.	3.30 (0.10-6.30)
Schneider *et al.*	2008	-	Germany	General	0-3	Cohort	5813	Outdoor	Fibrinogen	10°C Dec.	1.30 (0.20-2.40)
Wilker *et al.*	2012	-	USA	Cardiovascular	1-4	Cohort	100	Apparent	BNP	5°C Inc.	11.40 (1.20-22.50)
Sartini *et al.*	2017	1997–2000	UK	Aged	0	Cohort	10056	Outdoor	Vit-D	5°C Dec.	11.20 (1.0-20.40)
Chen *et al.*	2021	-	China	Hypertensive	0	Retrospective	112	Indoor	SBP	5°C Dec.	2.05 (0.60-3.40)
Umishio *et al.*	2019	2014–2017	Japan	General	0	Cross-sectional	1840	Indoor -Morning	SBP	5°C Dec.	4.1 (3.85-4.30)
Umishio *et al.*	2019	2014–2017	Japan	General	0	Cross-sectional	1840	Indoor -Evening	SBP	5°C Dec.	2.3 (2.15-2.45)
Sartini *et al.*	2017	1997–2000	UK	Aged	0	Cohort	10056	Outdoor	SBP	5°C Dec.	1.12 (0.60-.64)
Dasti *et al.*	2016	2003-2005	USA	Hypertensive	0	Cohort	819	Outdoor	SBP	5°C Dec.	9.0 (4.0-14)
Madaniyazi *et al.*	2016	2006-2011	China	General	3	Cohort	47,591	Outdoor	SBP	5°C Dec.	0.65 (0.5-0.8)
Madaniyazi *et al.*	2016	2006-2011	China	General	3	Cohort	47,591	Outdoor	SBP	5°C Inc.	3.0 (0.85-5.2)
Hurk *et al.*	2015	2007-2009	Netherlands	General	0	Cohort	101377	Outdoor	SBP	5°C Dec.	0.45 (0.35-0.55)
Yang *et al.*	2015	2004-2008	China	Cardiovascular	0	Baseline survey	23000	Outdoor	SBP	5°C Dec.	3.1 (2.85-3.35)
Giorgini *et al.*	2015	2003-2011	USA	Cardiovascular	4-6 days	Cohort	2078	Outdoor	SBP	5°C Dec.	18.0 (5.5-30.5)
Chen *et al.*	2015	2011-2012	China	Hypertensive	0	Cohort	50	Outdoor	SBP	5°C Dec.	0.95 (0.30-1.55)
Wu *et al.*	2015	2010-2011	China	General	0	Cohort	39	Outdoor	SBP	5°C Dec.	2.45 (1.45-3.4)
Saeki *et al.*	2014	2010-2013	Japan	Aged	0	Cohort	868	Indoor	SBP	5°C Dec.	1.1 (0.05-2.15)
Saeki *et al.*	2014	2010-2013	Japan	Aged	0	Cohort	868	Outdoor	SBP	5°C Dec.	1.45 (0.50-2.40)
Lanzinger *et al.*	2014	2007-2008	Germany	Type 2 Diabetic	0	Retrospective	30	Outdoor	SBP	5°C Dec.	5.0 (2.5-7.0)
Chen *et al.*	2013	1998-2001	China	Hypertensive	1-4 days	Cohort	1831	Outdoor	SBP	5°C Dec.	1.05 (1.05-1.1)
Kim et al	2012	2010	Korea	Aged	0	Cohort	20	Indoor	SBP	5°C Inc.	8.75 (-5.55-23.05)
Kim et al	2012	2010	Korea	Aged	0	Cohort	20	Outdoor	SBP	5°C Inc.	1.75 (-5.2-8.65)
Hampel *et al.*	2011	2002-2006	France	Pregnant	0	Cohort	1500	Outdoor	SBP	5°C Dec.	0.3 (0.05-0.55)
Kent *et al.*	2011	2003-2006	USA	General	0	Cohort	20,623	Outdoor	SBP	5°C Dec.	0.65 (0.40-0.85)
Barnett *et al.*	2007	1979-1997	16 countries	General	0	Cohort	115 434	Indoor	SBP	5°C Inc.	1.55 (0.9-2.2)
Barnett *et al.*	2007	1979-1997	16 countries	General	0	Cohort	115 434	Outdoor	SBP	5°C Inc.	4.55 (0.6-1.3)
Umishio *et al.*	2019	2014–2017	Japan	General	0	Cross-sectional	1840	Indoor -Morning	DBP	5°C Dec.	3.2 (2.95-3.50)
Umishio *et al.*	2019	2014–2017	Japan	General	0	Cross-sectional	1840	Indoor -Evening	DBP	5°C Dec.	2.1 (1.90-2.25)
Dasti *et al.*	2016	2003-2005	USA	Hypertensive		Cohort	819	Outdoor	DBP	5°C Dec.	0.5 (0.2-0.80)
Madaniyazi *et al.*	2016	2006-2011	China	General	3	Cohort	47,591	Outdoor	DBP	5°C Dec.	0.3 (0.25-0.40)
Madaniyazi *et al.*	2016	2006-2011	China	General	3	Cohort	47,591	Outdoor	DBP	5°C Dec.	0.65 (-0.65-1.95)
Hurk et al	2015	2007-2009	Netherlands	General	0	Cohort	101377	Outdoor	DBP	5°C Dec.	0.45 (0.35-0.55)
Giorgini *et al.*	2015	2003-2011	USA	Cardiovascular	4 to 6	Cohort	2078	Outdoor	DBP	5°C Dec.	12.5 (5.0-20.0)
Chen *et al.*	2015	2011-2012	China	Hypertensive	0	Cohort	50	Outdoor	DBP	5°C Dec.	0.60 (0.15-1.05)
Wu *et al.*	2015	2010-2011	China	General	0	Cohort	39	Outdoor	DBP	5°C Dec.	1.85 (1.15-2.55)
Chen *et al.*	2013	1998-2001	China	Hypertensive	1-4	Cohort	1831	Outdoor	DBP	5°C Dec.	0.70 (0.70-0.75)
Kim *et al.*	2012	2010	Korea	Aged	0	Cohort	20	Indoor	DBP	5°C Inc.	10.25 (0.25-20.25)
Kim et al	2012	2010	Korea	Aged	0	Cohort	20	Outdoor	DBP	5°C Inc.	1.0 (-4.15-6.10)
Kent *et al.*	2011	2003-2006	USA	General	0	Cohort	20,623	Outdoor	DBP	5°C Dec.	0.20 (0.10-0.35)

**Note: **^#^The data for SBP and DBP are given in mmHg (95% CI: min-max). **Abbreviations:** CRP, C-Reactive Protein; sVCAM, Soluble Cell Adhesion Molecule-1; sIAM, Soluble Intercellular Adhesion Molecule-1; TC, Total cholesterol; LDL, low-density lipoprotein cholesterol; IL-6, Interkleukin-6; BNP, B-type Natriuretic Peptide; SBP, Systolic blood pressure; DBP, Diastolic blood pressure; Pub, publication; Dec., Decrease; Inc., Increase.

## Data Availability

The raw data have been supplied as a Supplementary File.

## LIST OF ABBREVIATIONS

BNP: B-type Natriuretic Peptide
CRP: C-Reactive Protein
CV: Cardiovascular
DBP: Diastolic Blood Pressure
IL-6: Interleukin-6
LDL: Low-Density Lipoprotein
LDL-C: Low-Density Lipoprotein Cholesterol
NIHCR: National Institute for Health and Care Research
SBP: Systolic Blood Pressure
sCAM-1: Soluble Cell Adhesion Molecule-1
sIAM-1: Soluble Intercellular Adhesion Molecule-1
sVCAM: Soluble Cell Adhesion Molecule-1
TC: Total Cholesterol

## SUPPLEMENTARY MATERIAL

PRISMA checklist is available as supplementary material on the publisher’s website along with the published article. Supplementary material is available on the publisher’s website along with the published article.
